# Correction: Lipid signaling to membrane proteins: From second messengers to membrane domains and adapter-free endocytosis

**DOI:** 10.1085/jgp.20171187501122018c

**Published:** 2018-02-05

**Authors:** Donald W. Hilgemann, Gucan Dai, Anthony Collins, Vincenzo Lariccia, Simona Magi, Christine Deisl, Michael Fine

Volume 150, No. 2, February 2018. https://doi.org/10.1085/jgp.201711875

The authors regret that in the original version of this article, author Vincenzo Lariccia’s surname was mispelled.

All versions of this article have been corrected.

